# Dynamic changes of the respiratory microbiota and its relationship to fecal and blood microbiota in healthy young cats

**DOI:** 10.1371/journal.pone.0173818

**Published:** 2017-03-09

**Authors:** Aida I. Vientós-Plotts, Aaron C. Ericsson, Hansjorg Rindt, Megan E. Grobman, Amber Graham, Kaitlin Bishop, Leah A. Cohn, Carol R. Reinero

**Affiliations:** 1 College of Veterinary Medicine, University of Missouri, Columbia, Missouri, United States of America; 2 Department of Veterinary Medicine and Surgery, College of Veterinary Medicine, University of Missouri, Columbia, Missouri, United States of America; 3 Comparative Internal Medicine Laboratory, University of Missouri, Columbia, Missouri, United States of America; 4 University of Missouri Metagenomics Center, University of Missouri, Columbia, Missouri, United States of America; 5 Department of Veterinary Pathobiology, College of Veterinary Medicine, University of Missouri, Columbia, Missouri, United States of America; University of Illinois at Urbana-Champaign, UNITED STATES

## Abstract

Advances in the field of metagenomics using culture-independent methods of microbial identification have allowed characterization of rich and diverse communities of bacteria in the lungs of healthy humans, mice, dogs, sheep and pigs. These data challenge the long held belief that the lungs are sterile and microbial colonization is synonymous with pathology. Studies in humans and animals demonstrate differences in the composition of airway microbiota in health versus disease suggesting respiratory dysbiosis occurs. Using 16S rRNA amplicon sequencing of DNA extracted from rectal and oropharyngeal (OP) swabs, bronchoalveolar lavage fluid (BALF), and blood, our objective was to characterize the fecal, OP, blood, and lower airway microbiota over time in healthy cats. This work in healthy cats, a species in which a respiratory microbiota has not yet been characterized, sets the stage for future studies in feline asthma in which cats serve as a comparative and translational model for humans. Fecal, OP and BALF samples were collected from six healthy research cats at day 0, week 2, and week 10; blood was collected at week 10. DNA was extracted, amplified via PCR, and sequenced using the Illumina MiSeq platform. Representative operational taxonomic units (OTUs) were identified and microbial richness and diversity were assessed. Principal component analysis (PCA) was used to visualize relatedness of samples and PERMANOVA was used to test for significant differences in microbial community composition. Fecal and OP swabs provided abundant DNA yielding a mean±SEM of 65,653±6,145 and 20,6323±4,360 sequences per sample, respectively while BALF and blood samples had lower coverage (1,489±430 and 269±18 sequences per sample, respectively). Oropharyngeal and fecal swabs were significantly richer than BALF (mean number OTUs 93, 88 and 36, respectively; *p* < 0.001) with no significant difference (*p* = 0.180) in richness between time points. PCA revealed site-specific microbial communities in the feces, and upper and lower airways. In comparison, blood had an apparent compositional similarity with BALF with regard to a few dominant taxa, but shared more OTUs with feces. Samples clustered more by time than by individual, with OP swabs having subjectively greater variation than other samples. In summary, healthy cats have a rich and distinct lower airway microbiome with dynamic bacterial populations. The microbiome is likely to be altered by factors such as age, environmental influences, and disease states. Further data are necessary to determine how the distinct feline microbiomes from the upper and lower airways, feces and blood are established and evolve. These data are relevant for comparisons between healthy cats and cats with respiratory disease.

## Introduction

The Human Microbiome Project provided a platform for the study of site-specific microbial communities by using DNA-based sequencing to characterize resident bacterial populations. The gastrointestinal microbiota has been the most studied site thus far, and these studies continue to provide evidence of the influence that these complex microbial communities have on host health and disease. Early studies of site-specific microbial communities did not include the respiratory tract, due to the belief that the human lungs were sterile, and that organisms isolated from the lower airways were indicative of disease or bacterial translocation from the upper airways. With the help of culture-independent techniques, it has been demonstrated that healthy human airways harbor complex microbial populations, sometimes referred to as the “core airway microbiota” [1]. Alterations in these microbial communities have been documented in a variety of inflammatory airway diseases in humans, including asthma, COPD [2, 3], and cystic fibrosis [4]. However, it is not clear what role these microbial communities may play in host health, and prevention or exacerbation of disease.

Most of the research pertaining to the airway microbiota has been conducted in humans, and knowledge about the composition of the airway microbiota in companion animals that share our environment is limited. Inflammatory airway disease is common in dogs and cats, in particular chronic bronchitis in both species and allergic asthma in cats. Recently, it was shown that healthy dogs have a rich and complex lower airway microbiota that is distinct from other sites including the upper airways and gastrointestinal tract [5]. These data in healthy dogs are critical for an understanding of the role of microbial communities in infectious and non-infectious inflammatory canine respiratory diseases. Cats have been proposed to be a comparative and translational model for allergic airway disease as both experimentally induced and spontaneous feline asthma share similar features to human allergic asthma [6, 7]. In humans, differences in the microbial composition between healthy and asthmatic airways have been reported [8–10]. To date, studies documenting the presence of and characterizing the healthy airway microbiota have not been published in cats.

The primary aim of this study was to document the presence of and characterize the respiratory microbiota in healthy cats. As a secondary objective, fecal and blood samples were also collected and analyzed to investigate possible correlations between the microbial communities found at each site. As it is speculated that the airway microbiome may be established as a result of microbial migration via microaspiration, inhalation of bacteria and direct mucosal dispersion[11], comparing the upper and lower airways and the fecal microbiota provide evidence that although there is overlap between the populations, each site is distinct. Like the lungs, until recently, the blood had also considered a sterile environment, however, it has also been shown to possess a microbiome[12]. In addition, it has been speculated that the blood microbiome may be a link between intestinal dysbiosis and other conditions like diabetes[13], cardiovascular diseases[14], liver fibrosis[15], and other inflammatory diseases[16]. By including the blood samples, we were able to characterize the circulating microbial DNA present in healthy cats, which may provide pilot data for future studies evaluating potential correlations between the blood and respiratory microbiomes in health and disease, in both human and veterinary patients.

## Materials and methods

### Ethics statement

All studies were performed in accordance with the Guide for the Use and Care of Laboratory Animals, and were approved by the University of Missouri Institutional Animal Care and Use Committee (MU IACUC protocol #7891).

### Cats

Cats were bred from a colony (Comparative Internal Medicine Laboratory, University of Missouri, Columbia MO), were all sexually intact and were aged < 1 year (25–35 weeks by end of study). Cats belonged to one of two litters: litter A consisted of 3 females and litter B consisted of 2 males and 1 female. At the start of the study, cats in litters A and B were 24, and 14 weeks old respectively. Cats were group housed by sex with 2 males and 4 females housed in separate, large runs with elevated platforms to climb, hanging hammocks, and a variety of enrichment toys. Cats were socialized by members of the research team. The average (± standard deviation) body weight was 3.0 ± 1.3 kg. Cats were transitioned to a commercial dry diet formulated for growth (Purina growth formula, St. Louis, MO) at 4 weeks old, and remained on this diet for the duration of the study. Access to food and clean drinking water were provided *ad libitum*. Prior to anesthesia for sample collection, cats were fasted for at least 12 hours to minimize the likelihood of aspiration. Cats were determined to be healthy by absence of respiratory clinical signs, a normal physical examination by a board-certified veterinary internal medicine specialist and lack of cytologic evidence of infection or inflammation from bronchoalveolar lavage fluid (BALF) samples. Euthanasia was not an endpoint of the study; all cats were subsequently adopted into private homes.

### Sample collection

Fecal swabs, oropharyngeal (OP) swabs, and BALF were collected at the beginning of the study (day 0), and at week 2, and week 10. Additionally, blood was collected on week 10. The cats were anesthetized with ketamine 4 mg/kg IV. After anesthetic induction but prior to intubation, a sterile cotton tip swab was inserted at a minimum of 4 cm rectally to obtain a fecal sample, while avoiding contact with the perianal area. A second moistened sterile swab was used to vigorously rub the caudodorsal aspect of the oropharynx with the mouth manually opened and tongue pulled forward, while avoiding contact with the oral cavity. The oropharyngeal and rectal swabs were each added to 5 mL of sterile saline and placed on ice for transport to the laboratory. Cats were intubated using a sterile 3.5 to 4 French endotracheal tube. To collect BALF, a sterile 8 French red rubber catheter was threaded through the sterile endotracheal tube until it was gently wedged in a lower airway. A single 20 ml aliquot of sterile saline was instilled, aspirated, and placed on ice. Four milliliters of whole blood was obtained by jugular venipuncture (site shaved of fur and prepared with ethanol) into sterile tubes with the anticoagulant EDTA.

Promptly after collection, fecal, OP, BALF, and blood samples were centrifuged to pellet bacterial cells. Supernatant was discarded and pellets were resuspended in 800 μL lysis buffer adapted from Yu et al. (4% sodium dodecyl sulfate, 50 mM EDTA, 500 mM NaCl, and 50 mM Tris-HCl pH 8.0) [17]. All samples were banked at -80°C until the end of the study, and DNA was extracted as a single batch.

### DNA extraction

DNA from feces, OP, and BALF was extracted using the bead beating plus column method as previously described [5]. DNA from blood was isolated from 4 mL of anti-coagulated whole blood. Cells were lysed by adding 40 mL of hypotonic ACK buffer (150 mM NH_4_Cl, 10 mM KHCO_3_, 1 mM EDTA pH 7.2) and were incubated at room temperature for 10 min. Samples were centrifuged at room temperature for 20 min at 2,500 ×g. This procedure was repeated once, the supernatant was discarded and 2 mL of lysis buffer (500 mM NaCl, 50 mM Tris-HCl, 50 mM EDTA, 4% SDS) with 0.1 mg/mL proteinase K were added. Samples were incubated at 60°C for 3 hours, followed by organic solvent extraction. After incubation with at 37°C for 30 min with 0.1 mg/mL RNase A, DNA was precipitated with ethanol, re-suspended in 0.5mL TE (10 mM Tris-HCl pH 8.2, 1 mM EDTA), samples were stored at -20°C until PCR and sequencing.

### 16S rRNA library preparation, sequencing and informatics

Library construction and sequencing was completed at the University of Missouri DNA Core facility as previously described [5]. Assembly, filtering, binning, and annotation of DNA sequences was performed at the MU Informatics Research Core Facility as previously reported [5] with the exception that principal component analysis of 1/4 root-transformed data was performed using Past 3.13 (www.folk.uio.no/ohammer/past/).

### Statistical snalysis

Statistical analysis was performed using Sigma Plot 12.3 (Systat Software Inc., Carlsbad, CA). Differences between sample collection sites in DNA yield, coverage, richness and relative abundance were determined using repeated measures ANOVA on ranks with post hoc comparisons via Tukey test. Testing via PERMANOVA, using Past 3.13, was performed to identify differences in β-diversity between samples sites. Results were considered statistically significant for *p* values ≤ 0.05.

## Results

### Lower airways and blood represent sites of lower biomass than feces and upper airways

Fluorometric measurements employing a broad-range dsDNA kit revealed that DNA yields varied widely between the samples sites: fecal samples (mean ± SEM of 2.05 ± 0.48 ng/μL), OP swabs (0.63 ± 0.45 ng/μL), BALF (15.31 ± 3.61 ng/μL), and blood (52.06±12.27 ng/μL). Three out of 18 fecal samples and 6 out of 18 oropharyngeal samples were below the limit of detection. The samples that were below the limit of detection were not excluded from further analysis. In order to maximize the amount of loading template, all samples were concentrated to the minimum volume required for PCR.

**Fig 1:**
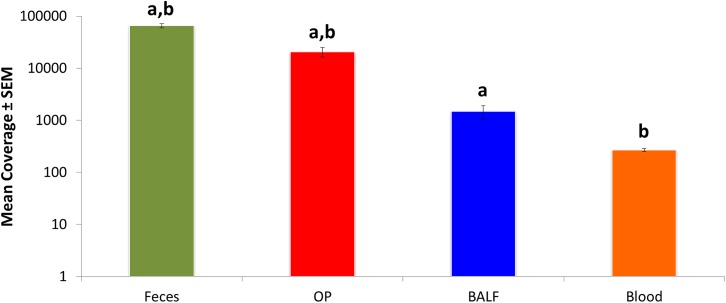
Mean ± standard error of the mean (SEM) coverage (number of sequences per sample) detected in DNA extracted from bronchoalveolar lavage (BALF), oropharyngeal swabs (OP), fecal swabs, and blood samples collected from 6 healthy young cats. Like letters indicate significant (*p* <0.05) differences.

Following amplification and sequencing, the total number of high-quality sequences detected (i.e., coverage) varied by site with fecal followed by OP samples yielding the highest number of sequences per sample (mean ± SEM of 65,653 ± 6,145 and 20,633 ± 4,360, respectively). As anticipated, BALF and blood had significantly lower coverage (1,489 ± 430 and 269 ± 18, respectively) compared to rectal and OP swabs (Fig 1).

For all samples, even those with lower biomass, and DNA yield below the limit of detection, consistent and interpretable data were obtained.

**Fig 2:**
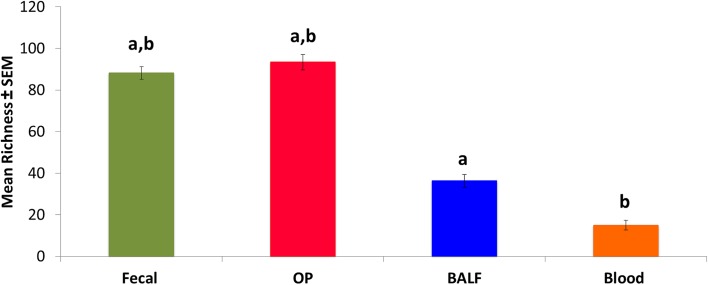
Mean ± standard error of the mean (SEM) richness (number of unique operational taxonomic units (OTUs) detected in the same samples. Like letters indicate significant (*p* <0.05) differences.

The richness of rectal and OP swabs was higher than that of BALF and blood; the mean number of operational taxonomic units (OTUs, groups of sequences sharing a minimum of 97% nucleotide identity) was 88, 93, 36 and 15, respectively (Fig 2). This was not unexpected as the lower airways and blood had lower DNA yields, which is consistent with sites with lower biomass. Dynamic changes in richness varied with site. In feces, there was no significant difference in richness between time points. In OP samples, there was significant increase in richness from week 6 to week 10 (mean OTUs 79 and 105, respectively; *p* = 0.012). In contrast, in BALF there was a significant decrease in richness from week 2 to week 10 (41 and 27, respectively; *p* = 0.043).

### *Proteobacteria* dominates healthy feline airways

**Fig 3:**
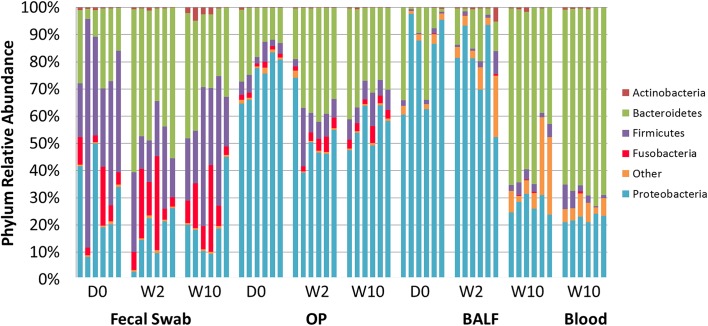
Relative abundance of all taxa detected in fecal, oropharyngeal swab (OP), bronchoalveolar lavage fluid (BAL), and blood collected at day 0, week 2, and week 10, annotated to the taxonomic level of phylum.

A distinct difference between microbial populations from the fecal and airway samples was detected at the taxonomic level of phylum (Fig 3). Consistent with previous reports in dogs [5] and humans [18], but in contrast to previous reports in cats [19]the predominant phyla detected in rectal swabs were *Bacteroidetes* (mean ± SEM relative abundance of 33.26 ± 3.11%), *Firmicutes* (31.55 ± 4.21%), *Proteobacteria* (21.70 ± 3.11%) and *Fusobacteria* (11.68 ± 2.40%). *Proteobacteria* was the most abundant phylum in the upper and lower airways (60.00 ± 3.23%, and 62.36 ± 6.72% in OP and BALF, respectively). Similar to rectal swabs, and in contrast to previous reports in humans [12] *Bacteroidetes* (67.60 ± 1.36%), and *Proteobacteria* (22.26 ± 0.56%), were the predominant phyla detected in blood samples.

From day 0 to week 10, there was a significant increase in the relative abundance of *Bacteroidetes* in feces (from 18.8± 4.33% to 33.05± 3.46%; *p* < 0.001), in OP (from 17.6±2.63% to 31.19±2.2%; *p* = 0.028) and in BALF (from 14.41±6.35% to 55.01±4.94%; *p* < 0.001). In contrast, there was a significant decrease in the relative abundance of *Proteobacteria* from day 0 to week 10 in OP (from 74.28±3.15% to 54.78±2.89%; *p* = 0.016), and in BALF (from 81.65±6.63% to 27.26±1.33%; *p* < 0.001). The relative abundance of the phyla *Firmicutes* and *Fusobacteria* did not significantly change over time.

**Fig 4:**
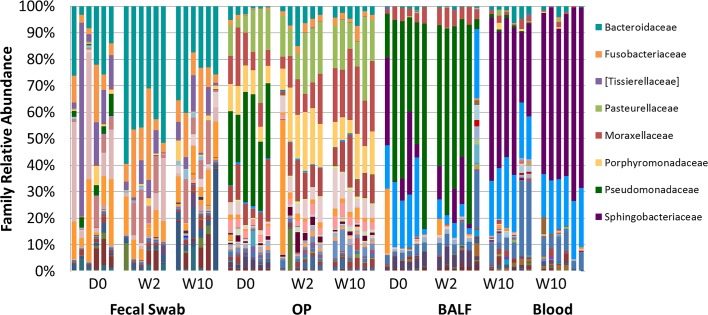
Relative abundance of all taxa detected in fecal, oropharyngeal swab (OP), bronchoalveolar lavage fluid (BALF) and blood collected at day 0, week 2, and week 10, annotated to the taxonomic level of family. The identity of dominant OTUs shown on the right.

Resolved to the taxonomic level of family (Fig 4), the differences in microbial composition between sites are highlighted, in particular when comparing rectal swabs with airway samples. The phylum *Bacteroidetes* was detected at greater relative abundance in feces, OP swabs, and blood samples relative to BALF, however it comprised different bacterial families and OTUs. In rectal swabs, the phylum *Bacteroidetes* consisted primarily of organisms in the *Bacteroidaceae* family (30.12 ± 3.60%). In OP swabs, this phylum was represented by the *Porphyromonadaceae* and *Paraprevotellaceae* families (12.45 ± 1.29% and 5.46 ± 0.87%, respectively). Although microbes in the phylum *Bacteroidetes* were present at much higher proportions in blood compared to BALF, the majority of the microbes in this phylum belonged to the family *Sphingobacteriaceae* (64.24±2.38% and 22.56±5.08% in blood and BALF, respectively).

Microbes in the phylum *Firmicutes* were represented by the families *Tissierellaceae* and *Lachnospiraceae* (10.31 ± 3.87% and 10.00 ± 1.87%, respectively), while the phylum *Fusobacteria* was dominated by the family *Fusobacteriaceae* (11.68 ± 2.40%). These families were mainly found in fecal samples and were detected at relatively low abundance in other sample sites. *Proteobacteria* were detected in fecal and blood samples with similar frequency; however, the DNA was annotated to different families. In rectal swabs, it was mostly represented by families *Campylobacteraceae* and *Enterobacteriaceae* (8.87 ± 3.32% and 7.09 ± 1.57%, respectively) while these taxa were rare in all other sample sites. In blood samples, *Bradyrhizobiaceae* (19.71 ± 2.30%) was the most abundant family in this phylum, and it was rarely found in fecal or OP samples. The airways were predominated by *Proteobacteria*, with *Pasteurellaceae*, *Moraxellaceae*, and *Pseudomonadaceae* being the most abundant families in the OP samples (15.99 ± 1.93%, 14.79 ± 1.73%, and 10.21 ± 3.69%, respectively). *Pseudomonadaceae* and *Bradyrhizobiaceae* were the most abundant families in BALF samples (34.24 ± 7.16% and 15.83 ± 2.18%, respectively). All taxa detected at greater than 0.50% mean relative abundance in at least one sample site are listed in Table 1.

**Table 1:** Relative abundance of all taxa detected at greater than 0.50% mean relative abundance in at least one sample site (feces, oropharyngeal swabs (OP), bronchoalveolar lavage (BAL) or blood), annotated to the level of phylum, family, and operational taxonomic unit (OTU). Data presented as mean ± standard error of the mean (SEM).

Phylum	Family	OTUs	Feces			OP			BAL			Blood		
*Actinobacteria*	*Corynebacteriaceae*	*Corynebacterium sp*.	0.75	±	0.23	0.01	±	0.00	0.06	±	0.03	0.00	±	0.00
*Bacteroidetes*	*[Paraprevotellaceae]*	*[Prevotella] sp*.	0.94	±	0.37	5.46	±	0.87	0.02	±	0.02	0.05	±	0.12
	*[Weeksellaceae]*	*Chryseobacterium sp*.	0.01	±	0.00	1.38	±	0.17	0.05	±	0.02	0.00	±	0.00
		Family [*Weeksellaceae*]	0.00	±	0.00	1.72	±	0.28	0.01	±	0.01	0.00	±	0.00
	*Bacteroidaceae*	*Bacteroides fragilis*	2.37	±	0.47	0.00	±	0.00	0.07	±	0.05	0.00	±	0.00
		*Bacteroides ovatus*	10.43	±	2.10	0.02	±	0.01	0.31	±	0.07	0.17	±	0.18
		*Bacteroides plebeius*	2.54	±	1.22	0.01	±	0.00	0.10	±	0.07	0.00	±	0.00
		*Bacteroides sp*.	14.44	±	2.56	4.23	±	0.81	0.89	±	0.27	1.26	±	1.58
	*Chitinophagaceae*	*Sediminibacterium sp*.	0.01	±	0.00	0.03	±	0.01	0.59	±	0.26	0.06	±	0.15
	*Flavobacteriaceae*	*Capnocytophaga sp*.	0.00	±	0.00	0.66	±	0.08	0.00	±	0.00	0.00	±	0.00
		Family *Flavobacteriaceae*	0.00	±	0.00	1.09	±	0.44	0.21	±	0.19	0.05	±	0.12
	*Porphyromonadaceae*	*Porphyromonas endodontalis*	0.00	±	0.00	2.15	±	0.33	0.02	±	0.02	0.00	±	0.00
		*Porphyromonas sp*.	1.15	±	0.54	10.16	±	1.14	0.09	±	0.08	0.10	±	0.25
	*Prevotellaceae*	*Prevotella copri*	0.57	±	0.34	0.01	±	0.00	0.04	±	0.02	0.00	±	0.00
	*Rikenellaceae*	*Family Rikenellaceae*	0.00	±	0.00	0.03	±	0.02	0.36	±	0.18	0.75	±	1.28
	S24-7	Family S24-7	0.04	±	0.01	0.02	±	0.00	0.82	±	0.28	0.79	±	0.79
	*Sphingobacteriaceae*	Family S*phingobacteriaceae*	0.01	±	0.01	0.00	±	0.00	22.43	±	5.10	64.25	±	5.84
*Firmicutes*	[*Tissierellaceae*]	*Anaerococcus sp*.	7.31	±	3.90	0.01	±	0.00	0.13	±	0.07	0.00	±	0.00
		*Peptoniphilus sp*.	2.07	±	0.59	0.00	±	0.00	0.08	±	0.06	0.00	±	0.00
	*Bacillaceae*	*Geobacillus sp*.	0.00	±	0.00	0.01	±	0.01	0.14	±	0.07	1.32	±	2.50
	*Erysipelotrichaceae*	*Family Erysipelotrichaceae*	0.02	±	0.01	0.54	±	0.10	0.02	±	0.02	0.00	±	0.00
	*Lachnospiraceae*	*[Ruminococcus] gnavus*	0.62	±	0.16	0.00	±	0.00	0.01	±	0.01	0.00	±	0.00
		*[Ruminococcus] sp*.	0.50	±	0.16	0.00	±	0.00	0.00	±	0.00	0.00	±	0.00
		*Dorea sp*.	0.50	±	0.19	0.00	±	0.00	0.01	±	0.01	0.00	±	0.00
		Family *Lachnospiraceae*	0.94	±	0.21	1.56	±	0.23	0.06	±	0.02	0.22	±	0.35
		*Roseburia sp*.	6.86	±	1.91	0.01	±	0.00	0.00	±	0.00	0.00	±	0.00
	Order C*lostridiales*	Order *Clostridiales*	0.43	±	0.12	0.13	±	0.06	0.44	±	0.15	0.70	±	1.29
	*Peptostreptococcaceae*	Family Peptostreptococcaceae	0.02	±	0.01	1.28	±	0.21	0.00	±	0.00	0.00	±	0.00
		*Peptostreptococcus sp*.	0.98	±	0.21	0.01	±	0.01	0.01	±	0.01	0.00	±	0.00
	*Ruminococcaceae*	*Faecalibacterium prausnitzii*	0.55	±	0.42	0.00	±	0.00	0.04	±	0.02	0.00	±	0.00
		*Family Ruminococcaceae*	0.83	±	0.16	0.01	±	0.00	0.08	±	0.04	0.11	±	0.27
		*Ruminococcus sp*.	0.55	±	0.07	0.00	±	0.00	0.00	±	0.00	0.00	±	0.00
	*Staphylococcaceae*	*Staphylococcus sp*.	1.29	±	0.59	0.92	±	0.87	0.19	±	0.08	0.30	±	0.25
	*Streptococcaceae*	*Streptococcus sp*.	3.59	±	1.02	0.27	±	0.05	0.37	±	0.11	0.29	±	0.46
	*Veillonellaceae*	*Megasphaera sp*.	0.63	±	0.26	0.00	±	0.00	0.03	±	0.02	0.00	±	0.00
*Fusobacteria*	*Fusobacteriaceae*	*Fusobacterium sp*.	11.68	±	2.40	2.04	±	0.32	0.12	±	0.05	0.15	±	0.37
*Proteobacteria*	*Alcaligenaceae*	*Kerstersia gyiorum*	0.00	±	0.00	2.96	±	2.80	1.76	±	1.37	0.00	±	0.00
		*Sutterella sp*.	0.78	±	0.17	0.00	±	0.00	0.01	±	0.01	0.00	±	0.00
	*Bradyrhizobiaceae*	*Family Bradyrhizobiaceae*	0.06	±	0.02	0.40	±	0.06	15.83	±	2.18	19.71	±	2.30
	*Burkholderiaceae*	*Lautropia sp*.	0.00	±	0.00	0.78	±	0.15	0.00	±	0.00	0.00	±	0.00
	*Campylobacteraceae*	*Campylobacter sp*.	8.87	±	3.32	0.19	±	0.03	0.09	±	0.05	0.00	±	0.00
	*Cardiobacteriaceae*	Family *Cardiobacteriaceae*	0.00	±	0.00	1.55	±	0.36	0.02	±	0.02	0.00	±	0.00
	*Comamonadaceae*	*Delftia sp*.	0.03	±	0.01	0.16	±	0.06	0.68	±	0.16	0.00	±	0.00
	*Enterobacteriaceae*	Family *Enterobacteriaceae*	7.09	±	1.57	0.01	±	0.00	0.11	±	0.05	0.00	±	0.00
	*Methylobacteriaceae*	*Methylobacterium sp*.	0.00	±	0.00	0.00	±	0.00	0.09	±	0.03	0.99	±	0.89
	*Moraxellaceae*	*Acinetobacter johnsonii*	0.11	±	0.05	1.01	±	0.35	3.48	±	0.57	0.25	±	0.28
		*Enhydrobacter sp*.	0.00	±	0.00	3.04	±	0.77	0.06	±	0.05	0.15	±	0.37
		Family *Moraxellaceae*	0.00	±	0.00	0.99	±	0.17	0.00	±	0.00	0.00	±	0.00
		*Moraxella sp*.	0.01	±	0.00	9.74	±	1.42	0.28	±	0.17	0.15	±	0.37
	*Neisseriaceae*	*Conchiformibius kuhniae*	0.02	±	0.01	8.61	±	1.23	0.10	±	0.08	0.10	±	0.25
		Family *Neisseriaceae*	0.00	±	0.00	1.16	±	0.14	0.03	±	0.03	0.00	±	0.00
	*Oxalobacteraceae*	Family *Oxalobacteraceae*	0.00	±	0.00	0.00	±	0.00	0.02	±	0.01	0.00	±	0.00
	*Pasteurellaceae*	*Actinobacillus sp*.	0.00	±	0.00	0.77	±	0.40	0.04	±	0.02	0.00	±	0.00
		Family *Pasteurellaceae*	0.05	±	0.03	11.77	±	2.05	0.12	±	0.10	0.05	±	0.12
		*Pasteurella multocida*	0.00	±	0.00	3.45	±	0.67	0.00	±	0.00	0.10	±	0.25
	*Pseudomonadaceae*	*Pseudomonas sp*.	1.18	±	0.52	10.13	±	3.67	33.90	±	7.12	0.00	±	0.00
	*Sphingomonadaceae*	*Sphingobium sp*.	0.09	±	0.04	0.76	±	0.29	2.51	±	0.51	0.00	±	0.00
	*Succinivibrionaceae*	*Anaerobiospirillum sp*.	2.87	±	1.96	0.00	±	0.00	0.01	±	0.01	0.00	±	0.00
	*Xanthomonadaceae*	Family *Xanthomonadaceae*	0.03	±	0.01	0.36	±	0.07	0.67	±	0.14	0.00	±	0.00
SR1	Phylum SR1	Phylum SR1	0.00	±	0.00	1.03	±	0.29	0.00	±	0.00	0.00	±	0.00

### Healthy airways have dynamic changes in relative abundance of key families within the phyla *Proteobacteria* and *Bacteroidetes*

The fecal microbiome was relatively stable over the study period. The only significant changes in the relative abundance of microbial families were increases in *Paraprevotellaceae*, *Pseudomonadaceae*, and *Alcaligenaceae*, none of which ever exceeded 2.5% of the total population. In OP samples, changes over time included a decrease in relative abundance of *Pseudomonadaceae* from day 0 to week 10 (30.57 ± 3.84% to 0.05 ± 0.01%; *p* < 0.001) and an increase in the relative abundance of family *Moraxellaceae* from day 0 to week 10 (11.15 ± 2.4% to 22.34 ± 1.80%; *p* = 0.008). Significant changes in the lower airway microbiota occurred between week 2 and week 10, when there was a significant decrease in the relative abundance of *Pseudomonadaceae* (from 52.64 ± 10% to 0.08 ± 0.01%; *p* = 0.005) with a concurrent increase in the relative abundance of *Sphingobacteriaceae* (from 7.5 ± 3.19% to 47.2 ± 5.4%; *p* < 0.001).

### Diversity of microbial communities detected at each sample site are significantly different

**Fig 5:**
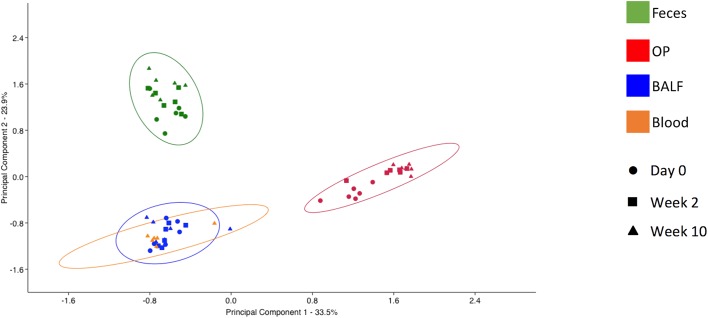
β-diversity as shown via principal component analysis. Unweighted principal component analysis of samples from all four sample sites (feces, BALF, OP and blood) PC1 versus PC2; legends at right. The ellipses represent 95% intervals.

**Fig 6:**
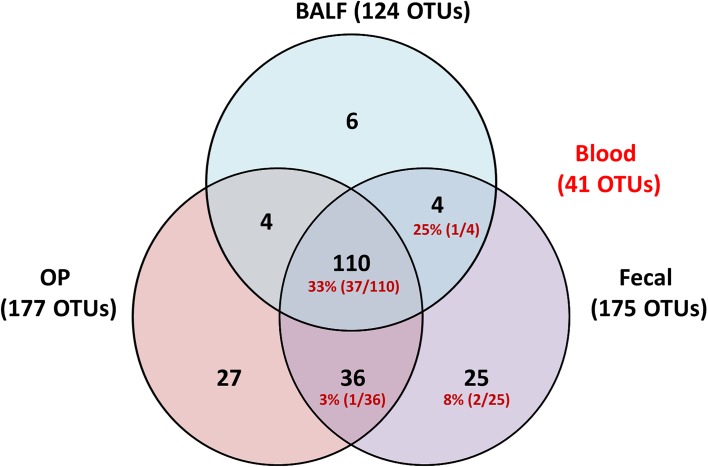
Venn diagram showing the distribution of operational taxonomic units (OTUs) detected in at least one bronchoalveolar lavage (BALF), oropharyngeal swab (OP), or fecal swab of healthy cats (n = 6) at one of three time points. Numbers in red indicate the number of OTUs from within that group that were also detected in blood.

Principal component analysis (PCA) was used to assess the β-diversity of the bacterial populations found at each site. When samples from all sites were included in the analysis, principal component 1 (PC1), accounting for 33.51% of the variation among the samples, showed complete separation of the fecal and OP microbial populations from BALF and blood (Fig 5). Interestingly, blood and BALF samples clustered closely together with regard to the first two principal components, reflecting the similarity seen in the stacked bar charts. Testing for differences in community structure between sample sites and time-points was performed via PERMANOVA. Two-way PERMANOVA was performed initially to determine the presence or strength of main effects due to either fixed variable; significant differences were detected associated with both sample site and date of collection (*p* = 0.0001; F = 32.26 and 9.62 respectively). Additionally, a significant, albeit weak, interaction was detected between variables (*p* = 0.0088; F = -0.23). To address those interactions and provide pairwise comparisons, a one-way PERMANOVA was performed. Considering first the differences within sample site and between time-point, the OP swabs differed most from time-point to time-point, and the fecal samples differed the least. Specifically, the OP swabs at any given time of collection differed significantly from the other two collections, with F values ranging from 2.20 to 10.58) (Table 2). Conversely, the composition of fecal samples only differed between the day 0 and week 10 samples and the strength of that difference was low (*p* = 0.04; F = 2.03). As predicted based on the stacked bar charts (Fig 4), the week 10 BALF samples differed significantly from the first two samples, but day 0 did not differ from week 2. Considering the differences between sample sites, significant differences were detected between all pairwise comparisons across sample sites, regardless of when the samples were collected. These data indicate that the microbial communities detected in each sample site are significantly different and that the airway communities may be more dynamic over time than the fecal microbiota. Additionally, it should be noted that while the scant DNA detected in blood samples appeared quite similar in composition to that identified in the final BALF sample, statistical analysis detected differences between these two sites. A subjective comparison of the OTUs found in at least one sample from each site provides further evidence of the relationship between the bacteria detected in the blood relative to the respiratory and fecal samples (Fig 6). Specifically, all OTUs detected in the blood were also detected in at least one fecal sample.

**Table 2:** p values (upper right) and F values (lower left) generated from one-way PERMANOVA of Bray-Curtis similarity indices between microbial DNA detected in bronchoalveolar lavage samples (BALF), oropharyngeal swabs (OP), rectal swabs (Feces), and blood collected at day 0 (D0), week 2 (W2), or week 10 (W10) from healthy adult cats (n = 6).

		BALF			OP			Feces			Blood
		D0	W2	W10	D0	W2	W10	D0	W2	W10	W10
BALF	D0		0.5643	**0.0022**	**0.0019**	**0.0026**	**0.0027**	**0.0025**	**0.002**	**0.0019**	**0.002**
	W2	0.728		**0.0017**	**0.0015**	**0.0023**	**0.0019**	**0.0025**	**0.0023**	**0.0028**	**0.0022**
	W10	15.1	15.99		**0.0032**	**0.0014**	**0.0023**	**0.0026**	**0.0024**	**0.0017**	**0.0184**
OP	D0	11.02	8.89	37.8		**0.0032**	**0.003**	**0.0024**	**0.0024**	**0.0021**	**0.0029**
	W2	27.3	22.18	32.72	7.639		**0.0071**	**0.002**	**0.002**	**0.0022**	**0.003**
	W10	34.45	26.98	42.94	10.58	2.197		**0.0017**	**0.002**	**0.0022**	**0.0019**
Feces	D0	12.98	11.11	15.66	12.52	11.31	13.59		0.0659	**0.0404**	**0.003**
	W2	19.96	16.73	22.59	20.3	15.32	19.47	2.035		0.3298	**0.002**
	W10	18.33	15.35	20.16	17.49	12.7	16.01	2.027	1.066		**0.0019**
Blood	W10	25.11	24.63	3	64.32	50.62	75.07	21.03	31.45	27.74	

## Discussion

To the authors’ knowledge, the current data provide the first evidence of a rich and dynamic lower respiratory tract microbiota in healthy cats. Previously, standard culture techniques with isolation of low numbers of bacteria from healthy cat airways has led to speculation that in the absence of lower airway inflammation, cats harbored limited types and small numbers of microbes, likely because of aspiration of oropharyngeal secretions [20, 21]. In the current study, the gastrointestinal tract, upper airways (OP), and lower airways (BALF) each have unique microbial communities, while microbial OTUs detected in blood samples overlapped completely with those found in the gastrointestinal tract. While there was some degree of regional continuity with roughly 92% of OTUs in the BALF being present in the OP samples (Fig 6), bacterial communities as assessed by diversity indices suggested BALF and OP populations were completely separated and thus, distinct. This likely represents adaptation to unique niches.

Different regions of the body have distinct, dynamic microbial communities. Each community within the body varies in structure depending on the location [22]. The gastrointestinal tract is one of the most complex ecosystems studied, and resident organisms provide a defense mechanism against colonization with pathogenic microbes, aid in nutrient metabolism and immunomodulation. In turn, the gastrointestinal tract provides the resident organisms a stable, nutrient rich environment [23]. It is this symbiotic relationship that helps maintain homeostasis in healthy individuals [24]. Alterations in that intricate balance within the microbial community can occur secondary to multiple factors, including environmental influences, exposure to pathogenic organisms, and the use of medications including antibiotics. Aberrant alterations in relative abundance or diversity of the microbial community members, particularly those with deleterious effects on health, are referred to as dysbiosis. The changes leading to dysbiosis may vary depending on the ecosystem. For example, in the gastrointestinal tract high biodiversity is associated with health and decreases in diversity are reported in disease, whereas in the vagina the opposite is true [25, 26].

In this population of cats the predominant phyla detected in rectal swabs were *Bacteroidetes* (mean ± SEM relative abundance of 33.26 ± 3.11%), *Firmicutes* (31.55 ± 4.21%), this is in contrast to previous studies in healthy cats. In one report [27], the majority of OTUs (87.3%) pertained to the phylum *Firmicutes* whereas only 2.4% were in the phylum Bacteroidetes. Another study[28] reported that *Firmicutes* was the most abundant phylum in cats (92%) and only 0.45% of the OTUs identified belonged to the phylum *Bacteroidetes*. Both of these studies sampled feces from a heterogeneous population of pet cats that lived in different environments, had variety of diet and a wide age range. Therefore, this discrepancy may be due to the differences in populations, the population in this study consisted of cats in similar age range, and stable environments and diet. Another point to consider is that a study[19] that evaluated microbial diversity along the gastrointestinal tract of healthy cats identified that the majority of OTUs in the phylum *Bacteroidetes* were isolated from the ileum and colon (13% and 50% respectively), followed by 5% in the rectum. Therefore, the rectal swabs may be more representative of the microbiota in the colon than evacuated fecal material.

The presence and characterization of the lower airway microbiome in humans has only recently become the focus of research. Prior to the use of culture-independent techniques, microbial populations identified in the lower airways were considered to be associated with disease or contamination from the upper airways. Recent studies comparing the upper and lower airway microbiota in humans have concluded that a distinct microbiota is present at each site [29–32]. The basis of initial colonization of the airways is poorly understood, however potential sources include uterine-placental environment; the maternal birth canal; and intestinal, dermal, and other environmental organisms introduced by inhalation and microaspiration [33]. Even with low biomass, the lung microbiome can contribute to the state of health and is susceptible to derangements from external factors including systemic (e.g., antibiotics) and inhaled (e.g., cigarette smoke, pollutants, etc.) substances [31, 34].

In the respiratory tract, dysbiosis has been described in asthma, COPD, and cystic fibrosis, and has been noted in comparisons between smokers and non–smokers [10, 29, 35, 36]. Loss of beneficial microorganisms, expansion of potentially harmful or pathogenic organisms and loss of overall diversity have all been described as contributing factors to dysbiosis [37]. Characterization and understanding of changes to the core microbiome that occur in disease have led to questions regarding potential opportunities for therapeutic intervention [38]. Further studies investigating whether or not restoration of a “health-associated” microbial community could represent an alternative treatment approach in respiratory disease, are warranted.

Knowledge of a healthy airway microbiome sets the stage for identification of states of dysbiosis with feline respiratory diseases relevant to human health. Cats are the only animal species that naturally develop a condition associated with eosinophilic airway inflammation, airway hyperresponsiveness and airway remodeling similar to allergic asthma in humans [7]. Although some differences exist, humans and cats share many anatomic, physiologic and immunologic features. Environmental allergens have been implicated in both feline and human asthma. With this knowledge, a model consisting of sensitization and challenge of cats with house dust mite allergen or Bermuda grass allergen and development of an asthmatic phenotype has been used investigate human and feline asthma [39]. As humans and pet cats share their environment, pet cats are susceptible to similar diseases such as asthma, making them an excellent pre-clinical model [7].

In human adults, the most abundant phyla detected in the lower airways are *Bacteroidetes* (*Prevotella* and *Porphyromonas spp*.) and *Firmicutes* (*Veillonella* and *Streptococus spp*.); organisms belonging to the phylum *Proteobacteria* (*Pseudomonas*, *Acinetobacter*) were less abundant [1, 29, 31, 40, 41]. Although these taxa were identified in all feline BALF samples throughout the study period, their relative abundance at the level of phyla was significantly different. Samples from day 0 and week 2 were predominated by *Proteobacteria*. However, a significant decrease in this phylum with concurrent increase in *Bacteroidetes* was observed from week 2 to week 10. These changes may have been associated with maturation. The cats in this study were at least 6 months of age at the conclusion of the study; this is the age at which cats reach sexual maturity. Similar changes in relative abundance at the level of phyla of murine and human neonatal respiratory microbiomes have been reported [42, 43]. A longitudinal study in children suggests that there may be a transition of respiratory microbial communities toward a more adult-like composition that takes place over the first two years of life [44].

The current study was relatively small and samples were obtained over a period of 10 weeks, therefore it is possible that the airway microbiome was still changing as a function of maturation. The small sample size does not provide enough power [45] to determine the significance of the changes in microbial community composition over time. However, the aim of this study was to characterize the microbial composition, not to compare it over time. Although the frequency of performing lavage has not been associated with changes in cell numbers or types over time in cats, [46] it is still possible that repeated lavage might have influenced a change in microbial community composition over time. Additionally, environmental factors such as diet, water, air quality and housing could play a role in alterations of the bacterial composition of the airways. However, since the cats were in a controlled environment, it is less likely that these factors may have influenced the changes observed.

The presence of bacterial 16S rRNA in blood was first documented in healthy humans in 2001 [16, 47]. A recent report described that the blood microbiome in healthy adult humans was significantly different than the gastrointestinal microbiome, as it was mostly composed of taxa from the phylum *Proteobacteria* with minimal representation of taxa from the *Bacteroidetes* and *Firmicutes [12]*. In our study, all of the OTUs identified in the blood were also found in the fecal swabs. While the role of the blood microbiome in health and disease remains to be determined, it has been hypothesized that it may be of importance in the development of the gut-brain axis, and the gut-lung axis [48–51]. Further studies are needed to better understand if the blood microbiome may provide means for immunomodulation, diagnosis, or therapeutic interventions in systemic disease.

## Conclusion

Until recently, respiratory microbiology has emphasized that bacteria cultured from the lung are pathogens and are implicated in disease. This study was the first to document the existence of a rich and diverse airway microbiota in healthy cats. Bacterial communities may be dynamic depending on the age and environment of an individual. Advances in metagenomics can help characterize a dynamic respiratory ecosystem that can be disrupted in disease, altering homeostasis between host and microbiota. A better understanding of the differences in microbial communities in healthy versus inflammatory airways sets the stage for future studies to determine if the microbiota can be modulated to attenuate disease and/or restore immunologic tolerance.

## Abbreviations

BALF: bronchoalveolar lavage fluid
OP: Oropharyngeal
OTU: operational taxonomic unit
PCA: principal component analysis
